# Application of the omics sciences to the study of *Naegleria fowleri*, *Acanthamoeba* spp., and *Balamuthia mandrillaris*: current status and future projections

**DOI:** 10.1051/parasite/2021033

**Published:** 2021-04-12

**Authors:** Libia Zulema Rodriguez-Anaya, Ángel Josué Félix-Sastré, Fernando Lares-Villa, Luis Fernando Lares-Jiménez, Jose Reyes Gonzalez-Galaviz

**Affiliations:** 1 CONACYT-Instituto Tecnológico de Sonora Ciudad Obregón 85000 Sonora México; 2 Departamento de Biotecnología y Ciencias Alimentarias, Instituto Tecnológico de Sonora Ciudad Obregón 85000 Sonora México; 3 Departamento de Ciencias Agronómicas y Veterinarias, Instituto Tecnológico de Sonora Ciudad Obregón 85000 Sonora México

**Keywords:** Free-living amoebas, Next generation sequencing, Genomic, Transcriptomic, Proteomic, Omics

## Abstract

In this review, we focus on the sequenced genomes of the pathogens *Naegleria fowleri*, *Acanthamoeba* spp. and *Balamuthia mandrillaris*, and the remarkable discoveries regarding the pathogenicity and genetic information of these organisms, using techniques related to the various omics branches like genomics, transcriptomics, and proteomics. Currently, novel data produced through comparative genomics analyses and both differential gene and protein expression in these free-living amoebas have allowed for breakthroughs to identify genes unique to *N. fowleri*, genes with active transcriptional activity, and their differential expression in conditions of modified virulence. Furthermore, orthologous genes of the various nuclear genomes within the *Naegleria* and *Acanthamoeba* genera have been clustered. The proteome of *B. mandrillaris* has been reconstructed through transcriptome data, and its mitochondrial genome structure has been thoroughly described with a unique characteristic that has come to light: a type I intron with the capacity of interrupting genes through its self-splicing ribozymes activity. With the integration of data derived from the diverse omic sciences, there is a potential approximation that reflects the molecular complexity required for the identification of virulence factors, as well as crucial information regarding the comprehension of the molecular mechanisms with which these interact. Altogether, these breakthroughs could contribute to radical advances in both the fields of therapy design and medical diagnosis in the foreseeable future.

## Abstract (fr)

Dans cette revue, l’accent est mis sur les génomes séquencés des agents pathogènes *Naegleria fowleri*, *Acanthamoeba* spp. et *Balamuthia mandrillaris*, et les découvertes remarquables concernant la pathogénicité et l’information génétique de ces organismes, en utilisant des techniques liées aux diverses branches de l’omique comme la génomique, la transcriptomique et la protéomique. Actuellement, de nouvelles données produites par des analyses génomiques comparatives et l’expression différentielle des gènes et des protéines dans ces amibes libres ont permis des percées pour identifier des gènes uniques à *N. fowleri*, des gènes avec une activité transcriptionnelle active et leur expression différentielle dans des conditions de virulence modifiée. En outre, les gènes orthologues des divers génomes nucléaires des genres *Naegleria* et *Acanthamoeba* ont été regroupés. Le protéome de *B. mandrillaris* a été reconstruit grâce aux données du transcriptome, et la structure de son génome mitochondrial décrite de manière détaillée, mettant ainsi une caractéristique unique à jour : un intron de type I avec la capacité d’interrompre les gènes par son activité d’auto-épissage des ribozymes. Avec l’intégration des données issues des diverses sciences omiques, il existe une approximation potentielle qui reflète la complexité moléculaire requise pour l’identification des facteurs de virulence, ainsi que des informations cruciales concernant la compréhension des mécanismes moléculaires avec lesquels ceux-ci interagissent. Dans l’ensemble, ces percées pourraient contribuer à des progrès notables à la fois dans les domaines de la conception de la thérapie et du diagnostic médical dans un avenir proche.

## Introduction

Unlike classic molecular techniques, those involving omics science generate results at a complete level of the organism, depending on the approach taken, ranging from genes (genomics), to transcripts (transcriptomics), proteins (proteomics), and metabolites (metabolomics) [57]. With genomics, it is possible to identify genes globally, either completely or by identifying known protein domains that allow progress in a possible functional annotation [30]. The approach can also be used by comparing genomes between species to identify common proteins or, better yet, find differences between species with different levels of virulence to determine critical factors involved in these phenotypes or even patterns of evolution [31, 66]. Similarly, transcriptomics enables us to identify all the transcripts present in an organism at a given moment, be they RNAs involved in regulatory mechanisms (siRNA, miRNA, lncRNA, etc.) or RNAs that encode proteins, which is ideal for characterizing the gene expression under different conditions [66]. Proteomics is used to identify and/or compare the production of proteins in a particular condition and classifying them according to their function and location. These may also have their applications in comparative analysis, mainly applied to the recognition of overexpression or underexpression of transcripts and proteins [66].

Omics is a powerful set of tools for studying pathogens like amoebas, which are protist that can be located within two groups: parasitic and free-living. Those considered parasitic depend on a host to survive, while those that are free-living can subsist on their own in nature [68]. Within the group of free-living amoebae (FLA), there are specimens capable of exercising parasitic activity and acting as pathogens for different organisms. Due to this ability to survive both endozoically and independently, they were given the term amphizoic amoebae [60]. Among these, species from three genera can act pathogenically, causing infections mainly of the central nervous system: *Balamuthia mandrillaris*, *Naegleria fowleri*, and *Acanthamoeba* spp., each of them having a different pathogenesis mechanism causing different diseases. *Naegleria fowleri* causes primary amebic meningoencephalitis (PAM), *Acanthamoeba* spp. are responsible for amoebic keratitis and granulomatous amoebic encephalitis (GAE). Additionally, *B. mandrillaris* causes GAE and skin lesions [45, 46]. In the first instance, the pathogenesis mechanisms of these FLA were unknown, since initially, the only certainty was that amoebae reached the central nervous system through the olfactory neuroepithelium or through the blood in the case of *Acanthamoeba* spp. and *B. mandrillaris* [59, 67]. Since then, multiple studies have been done on these species to determine how they attack the central nervous system and what treatments can be effective in fighting them [20, 55, 56, 71]. Today, information on these pathogens is abundant since various mechanisms involved in immune response cascades related to their pathogenicity have already been identified [2, 53, 62]. However, there are still mechanisms to decipher in this regard since data of a genomic, transcriptomic, or proteomic nature are still scarce compared to other pathogens. Likewise, the purpose falls on informing about the structural genomics and functional genomics of FLA and all the remarkable discoveries regarding the pathogenomics of these microorganisms from techniques related to the various omic branches.

## Omics applications in free-living amoebae

### *Naegleria fowleri*

Given the progress in DNA sequencing technologies, generation of and access to genomic data allow us to perform whole comparative analysis and search for important evolution mechanisms. Therefore, current technologies and bioinformatics workflows can allow us to associate sequence variation with the pathogenesis and virulence of FLA. The comparative analysis of genomes of different strains can provide us with information on the genetic diversity and adaptation of the species. However, for these analyses to be successful, it is necessary to work with genomic data with flawless quality to reduce errors and artifacts in the comparison [27]. Due to the large increase in the number of genomes deposited in the databases, six quality categories for genomes have been described depending on their assembly, ranging from the Standard Draft to the Finished level or “Gold Standard,” where the latter refers to genomic sequences with an error rate of 1 per 100,000 base pairs and where repeated sequences and assembly errors have been resolved [10]. These high-quality sequences are recommended for all types of analysis that involve the comparison of genomes. In this context, we can mention that a few sequencing projects have been carried out for FLA, of which no amoebic genome has reached the Gold Standard level of assembly.

Currently, *N. fowleri* is the FLA with the most bioinformatic strategies regarding its structural and functional genomics. There are four sequenced genomes of *N. fowleri*, assembled at the scaffold level. The first one made is a hybrid assembly of the ATCC 30863 strain, which combines Illumina and Roche 454, carried out in 2014 [75]. This report presents a size of 29.6 Mb and 35.4% of guanine-cytosine content (GC), although this information is not reflected in its entry in the database, which instead shows a size of 27.8 Mb (GenBank assembly accession: GCA_000499105). In this report, approximately 17,252 open reading frames (ORFs) were predicted and compared with the RefSeq database through a BLASTp search, resulting in 16,021 matches, from which 7820 matched with gene ontology (GO) terms.

The second genome was sequenced from strain 30,894 with an Oxford Nanopore platform (GridION), whose initial assembly was improved with high-quality paired-end reads from Illumina. This genome has a size of 29.5 Mb and a GC percentage of approximately 36.9%. Similarly, it has repeated sequences of 6% and a total of 13,925 annotated genes. Of this genome, 208 proteins potentially secreted by the amoeba have been identified, where 20% were annotated with the term hydrolase activity and 10% with lipid/protein/ion binding function. On the other hand, other terms such as catalytic activity, enzyme regulation, and isomerase activity were identified in 10%, 3.8%, and 1%, respectively. Of these proteins, 27 were classified as serine proteases and 21 as lipid-degrading proteins. Moreover, three proteins similar to the autocrine proliferation repressor aprA and the countin-1 cell count complex of amoeba *Dictyostelium discoideum*, proteinase, and ribonuclease inhibitor proteins were identified. Furthermore, during this annotation process, 107 genes with coding potential for protein with unknown or uncharacterized functions were observed. Finally, with this genome, a phylogenomic analysis was carried out among the reported genomes of the genus *Naegleria*, placing the ATCC 30863 strain as the closest, followed by *N. lovaniensis*, while *N. gruberi* was found to be phylogenomically more distant [47]. In addition to the genomes described above, the sequences of *N. fowleri* strains “986” and “V212” were published in 2020, which will be described later [34].

Initially, *N. gruberi* was considered a candidate to carry out comparative genomics studies since it presented a broad characterization at the genomic level (GCA_000004985). This genome, unlike *N. fowleri*, has a size of approximately 41 Mb, of which 5.1% are repeated sequences and 57.8% code for proteins. Furthermore, it presents a GC percentage of 33%, very similar to that of *N. fowleri* [21]. Due to this, Herman et al. [35] carried out a comparative analysis between the *N. gruberi* genome and a 60 Kb nuclear genome segment of *N. fowleri* in order to find genes unique to *N. fowleri* and paralogs that grants it its particular pathogenic capabilities. Additionally, their mitochondrial genomes were compared. Although the mitochondrial genomes are highly similar in quantity and synteny, nuclear genomes are highly disparate and quite collinear. This comparative analysis showed approximately 31 putative ORFs in the 60 Kb segment of *N. fowleri*. Of these, 12 are homologous ORFs from other eukaryotic genomes, with domains of the Vps9 type, Hsp40, and ERV1; nine are specific to the genus *Naegleria,* and the remaining 10 do not have homologs identified in any eukaryotic genome. Although there is a possibility that some ORFs are poorly predicted, according to a recent study, they are more likely to be *N. fowleri* specific ORFs and emphasizes the importance of conducting molecular tests on them. Returning to the ORFs with identified homologs, it was observed that six of these encode functional proteins and that the rest contain recognizable conserved domains [34].

Due to the low synteny that *N. fowleri* presents with *N. gruberi*, the latter is no longer considered an ideal candidate for comparative genomic analysis. However, it has been reported that *N. lovaniensis* is a closer species phylogenomically and is now considered a better candidate for this type of analysis since it has a higher similarity with its rRNA sequences. On the one hand, the sequence of the small ribosomal subunit of *N. lovaniensis* only differs by 16 bp with the sequence of *N. fowleri,* and in the case of the 5.8S sequence, they are only distinguished by one base. Therefore, it is estimated that *N. fowleri* and *N. lovaniensis* come from a common ancestor and are the closest evolutionary species of *Naegleria* [40]. The *N. lovaniensis* genome was sequenced and assembled *de novo* in 2018 (GCA_003324165); 77% of the genome was coded with an approximate size of 30.8 Mb. Likewise, it presents GC content of 37%, a value quite similar to that of *N. fowleri* (35.4%) and disparate to *N. gruberi* (33%); since this value is slightly higher in *N. fowleri* and *N. lovaniensis,* it seems to be an allusion to their thermotolerance [35, 48]. Digging deeper into a recent genomic analysis, *N. lovaniensis* shares 9547 genes with *N. fowleri*, while *N. gruberi* only shares 5831 genes [48]. These results confirm the similarity tests carried out by Zysset-Burri et al. [75] in which only 32.1% of ORFs belonging to *N. fowleri* were aligned with the *N. gruberi* genome, although in the same study, it was proven that, despite the contrast in the genomic order, 78.2% of the ORFs coincided with genes from *N. gruberi* in a search with BLASTp. This emphasizes the need to focus on comparative studies between *N. fowleri* and *N. lovaniensis* due to the low genetic similarity with *N. gruberi*.

Bringing together all the predicted proteins of these *Naegleria* species into gene families gives a total of 8114 families, of which 2406 are shared between *N. fowleri* and *N. lovaniensis*. On the other hand, *N. fowleri* presents 323 specific families [48]. Through a gene ontology analysis, it was observed that these families are related to proteins of secretory processes, protein transport, and overexpression of the membrane component term. Furthermore, identity searches in BLAST of these proteins match with the protein families of Rab, Rho, and other proteins with catalytic cyclase domains of adenylate and guanylate [48]. This is related to a proteomic study carried out between a model of *N. fowleri* cultivated for high virulence compared to another cultivated for low virulence, in which the guanine exchange protein of Rho 28 is identified within the group of proteins belonging to that of high virulence. However, several homologs to structural and signal proteins downstream of integrins are identified. This is emphasized in Rho because it acts as a downstream signal regulator for integrin receptors and growth factors. Finally, Rho is described as a potential pathogenicity factor since, by performing a tissue invasion test in the presence of Rhosin, the invasion was reduced by 65% [39].

Burri et al. [8] discovered that when in a supplemented culture with liver hydrolyzate, trophozoites of *N. fowleri* become highly virulent, whereas those grown in PYNFH medium are attenuated in this aspect. Using this study model, Zysset-Burri et al. [75] conducted a comparative proteomic study to identify *N. fowleri* virulence factors. In the first instance, a visual comparison was performed employing 2D electrophoresis, using cofilin as a control protein site. Here, the overexpression of proteins already identified as pathogenic factors, such as HSP70, actins 1 and 2, and the membrane protein Mp2CL5, was verified in the high virulence proteome. However, cyclophilin was also identified as a potential pathogenicity factor as it was overexpressed in highly virulence trophozoites. In parallel, comparative genomics between pathogenic (*Entamoeba histolytica* strain HM-1: IMSS, *N. fowleri* ATCC 30863) and non-pathogenic amoebae (*Willaertia magna* c2c maky, *N. gruberi* NEG-M, *N. lovaniensis* ATCC 30569) reported the presence of homologs associated with the virulence of *N. fowleri* within the pangenome of *W. magna* c2c maky and *N. gruberi*, from which HSP70, Mp2CL5, actin, and Nf314 stand out [33]. However, the presence or absence of these genes is not enough evidence to relate them to pathogenicity or virulence. More advanced studies, like those we will mention further ahead, are necessary to elucidate these characteristics inherent to each organism.

For epidemiological approaches, genetic variations in *N. fowleri* have been identified, classifying this amoeba into eight genotypes unevenly distributed around the world. In Europe, types 8, 7, 6, 4, and 3 have been reported; in the USA, types 1, 2, and 3; in Mexico, type 2; in Asia types 2 and 3; and finally, in Japan and Oceania, only type 5 [41]. In order to distinguish genotypes, the length of the internal transcript spacer 1 (ITS1) sequence is mainly used as a basis, the nucleotide transition (C/T) of position 31 of the 5.8S rRNA sequence is also considered. Representative sequences of each genotype reported in GenBank can be consulted through the following access numbers: AJ132028, AJ132030, AY376149, FR875287, FR875288, X96563, X96562, and X96564 [74]. To date, updates have been developed regarding the genomic and transcriptomic characterization of *N. fowleri* using genotype 5 to obtain the genome of strain 986 with Illumina HiSeq 2000 to identify new pathogenicity factors [34].

Likewise, the genome sequencing of *N. fowleri* strain V212 was also carried out, for which the transcriptome was sequenced to enrich gene prediction. Finally, the virulence of the LEE strain was modified when passed through mice to obtain its transcriptome and identify the differentially expressed genes (DEG) (which are particularly important for the design of treatments) considering those with a LogFC ± 1, a lax FDR lower than 0.1 and lacking similarity to human genes. In this way, 315 DEGs were identified in the LEE strain passed by mice compared to the same strain grown in axenic medium, of which 208 are up-regulated and 107 down-regulated. Among the up-regulated, the most relevant were: actin, the precursor protein of Naegleriapores A and B; cathepsin A, GTPase Rab 32, the retromer component Vps 35, RhoGaP22, kinase PAK3, transcription factor RWP-RK, and a group of 28 proteases that comprise more than 10% of these genes. The most represented types of proteases in this group are cathepsin proteases, particularly the C01 subfamily, including cathepsins B, C, L, Z, and F. Notably, 90 of the up-regulated genes have no human orthologs, making these potential targets for treatment. Likewise, of the up-regulated genes, 40 are specific for the genus *Naegleria* and 11 specifics for *N. fowleri*. In the case of down-regulated ones, the genes found are mainly related to transcription and translation repression and signal transduction. There are 19 genes specific to *Naegleria* and 26 specifics to *N. fowleri*. However, these results are derived from using a lax FDR lower than 0.1; in case of using an FDR lower than 0.05 as usual, only 134 up-regulated genes would be counted, with 21 gender-specific genes and 7 *N. fowleri*-specific genes, as well as 64 down-regulated genes, and among these, ten gender-specific genes and 16 specifics to *N. fowleri*. Finally, it should be mentioned that in the comparative genomic-transcriptomic analysis carried out with these data, a total of 458 *N. fowleri*-specific genes shared by strains 986, V212, and LEE were identified, which are not present in *N. gruberi*, and from them a total of 390 lack known function [34]. For better visualization, in Table 1, we have summarized the main genes related to *N. fowleri* pathogenicity with the respective omic approach used for their identification.

**Table 1:** Relation of genes described with pathogenicity potential found in *N. fowleri*.

Pathogenicity-related genes	Gene function	Database accession	OMIC approach
Heat Shock Protein 70 (hsp70)	Cytotoxicity and proliferation related	GenBank AY684788	Comparative proteomics [75]
Actin 1	Attachment of amoebae to substrates and phagocytosis	GenBank M90311	Comparative proteomics [75], comparative genomics and transcriptomics [34]
Actin 2	Attachment of amoebae to substrates and phagocytosis	GenBank M90312	Comparative proteomics [75], comparative genomics and Transcriptomics [34]
Formin D	Regulate the formation of actin filaments	Uniprot accession number Q5TJ55	Comparative proteomics [75], comparative genomics and transcriptomics [34]
Severin	Actin-fragmenting and capping proteins	Uniprot accession number P10733	Comparative proteomics [75], comparative genomics and transcriptomics [34]
Villin-1	Multifunctional actin cytoskeleton regulating protein	Uniprot accession number Q3SZP7	Comparative proteomics [75], comparative genomics and transcriptomics [34]
Myosin II	Phagocytic processes	Uniprot accession number P08799	Comparative proteomics [75]
Membrane protein Mp2CL5	Contact-dependent pathogenesis	GenBank AY049749	Comparative proteomics [75]
Cyclophilin	Stimulation of pro-inflammatory signaling	GenBank XM_002681214	Comparative proteomics [75]
Apoptosis-linked gene-2-interacting protein X1 (AIP1)	Vesicular trafficking	Uniprot accession number P34552	Comparative proteomics [75]
Naegleriapore A	Vesicular trafficking/destroy target cells	Uniprot accession number Q9BKM2	Comparative proteomics [75], genomics [47], comparative genomics and transcriptomics [34]
Naegleriapore B	Vesicular trafficking/destroy target cells	Uniprot accession number Q9BKM1	Comparative proteomics [75], comparative genomics and transcriptomics [34]
Golgi-localized transmembrane protein HID-1	Vesicular trafficking/vesicular exocytosis	Uniprot accession number Q8IV36	Comparative proteomics [75]
Ras-related protein Rab-1	Vesicular trafficking/phagocytosis regulation	Uniprot accession number Q4UB16	Comparative proteomics [75]
Virulence-related protein Nf314	Protease	Uniprot accession number P42661	Genomics [47], comparative genomics and transcriptomics [34]
Heat Shock Protein 40 (hsp40) domain	Regulator of HSP70	Uniprot accession number A0A6A5BGT1	Genomics [47], comparative genomics [35]
Serine protease S81	Lysozyme activity	GenBank AAA96144.1	Comparative genomics and transcriptomics [34]
Rab GTPase Rab32	Endo-lysosomal trafficking gene	GenBank XP_002683044.1	Comparative genomics and transcriptomics [34]
Retromer component Vps35	Endo-lysosomal trafficking gene	GenBank NP_060676.2	Comparative genomics and transcriptomics [34]
Nfa1	Cell–cell adhesion factor	Uniprot accession number Q9NH76	Comparative genomics and transcriptomics [34]

The analysis of comparative genomics between non-pathogenic *Naegleria* spp. and *N. fowleri*, aided by the information obtained from the DEGs found within the modified virulence strains of *N. fowleri* mentioned above, is as an outstanding contribution to deciphering which genes are active during infection. First of all, exploiting the benefits of applying comparative genomics between pathogens and non-pathogens could lead to identification of the function of the annotated genes. This would be based on the sequences conserved within homologs with known function in other species [16]. Additionally, it permits the observation of genetic variation at different levels (genotype, species, genera), revealing evolutive relations. Subsequently, through the analysis of possible metabolic routes where genes previously revealed via transcriptomics interact with one another, more convincing conclusions about the virulence and pathogenicity of *N. fowleri* could be obtained [7]. Finally, those genes with a significant differential expression could be used to design an infection model, through the application of reverse genetics, to block the expression of candidate genes with virulence-related functions, and make specific function analyses for genes identified as unique to *N. fowleri* or for the design of targeted treatments.

**Figure 1:**
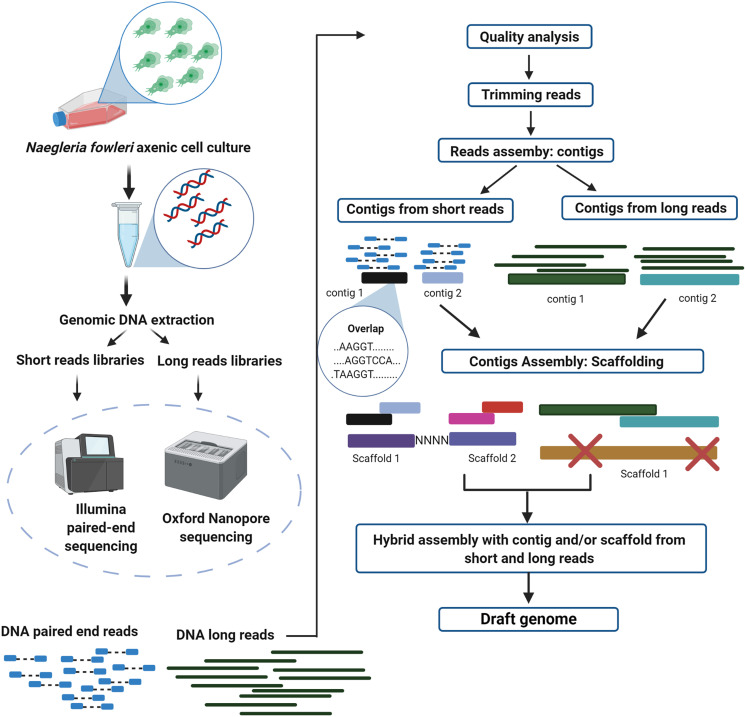
(a) Workflow for data management using short and long reads to obtain high-quality hybrid genome assembly. Advanced analyses are shown in Figure 1b. Created with BioRender.com. (b) Advanced analyses for the improvement of genome assembling, achieving accurate genetic identification through the use of different omic data (mainly genomic, transcriptomic, and proteomic), to characterize a phenotype of interest. Created with BioRender.com.

Describing the above, Table 2 summarizes the general information of the consulted genomes, and in Figures 1a and 1b, we display a proposal for advanced bioinformatic analyses, utilizing the improved *N. fowleri* genome reported by Liechti et al. [47], which has a hybrid genomic assembly approach. These types of assemblies are based on the use of sequence platforms such as Illumina, which produce high-quality readings that range from 50 to 300 bp. However, due to their short size, genomic assemblies with them result in highly fragmented genomes, which in turn prevent the resolution of large repeating regions [13]. By contrast, Oxford Nanopore Technology (ONT) produces large-sized reads that range between 500 bp and 2.3 Mbp [1]. The large readings manage to cover repeated regions, producing less fragmented or even completed genomes, with the only downside being that ONT has a high error rate. Therefore, a hybrid assembly is defined as a bioinformatic strategy implemented to solve the limitations of both technologies, Illumina and ONT, at the same time, while taking advantage of their strengths, solving Ilumina’s problem with repeated regions through ONT’s large reads, and solving ONT’s high error rate with Ilumina’s high-quality reads [13].

**Table 2:** Data from the assembled genomes of *Naegleria* spp.

Organism	Sequencing technology	Genome size (Mb)	%GC	ORFs	Strain	GenBank assembly accession	Data publication date	Reference
*N. fowleri*	Illumina HiSeq 2000 and Roche 454 – GS FLX	27.7	37	17,252	ATCC 30863	GCA_000499105	November 2013	[75]
*N. fowleri*	Illumina HiSeq and Roche 454 – GS FLX	27.7	36	12,677	V212	*PRJNA562275	August 2019	[34]
*N. fowleri*	Illumina – Hiseq 2000	27.5	36	11,599	986	GCA_902703645	February 2020	[34]
*N. fowleri*	Oxford Nanopore – GridION	29.5	36.9	13,925	ATCC 30894	GCA_008403515	September 2019	[47]
*N. gruberi*	Sanger	40.9	33	15,727	NEG-M ATCC 30224	GCA_000004985	January 2010	[21]
*N. lovaniensis*	PacBio – RSII	30.9	37	15,195	ATCC 30569	GCA_003324165	July 2018	[48]

* For strain V212, BioProject number indicated because authors did not indicate GenBank assembly accession.

### *Acanthamoeba* spp.

The *Acanthamoeba* genus is usually molecularly characterized into genotypes based on the small ribosomal subunit gene (SSU-18S rRNA) [44]. To date, 22 genotypes have been established: T1–T12 [70], T13 and T14 [37], T15 [36], T16 [15], T17 [58], T18 [64] T19 [50], T20 [24], T21 and T22 [72], geographically diversely distributed, so this amoeba has a presence on all continents [5]. For this reason, and since symptoms during infection vary between genotypes, genetic analyses have been done at the genomic level to identify the origin of these variations [23]. *Acanthamoeba castellanii* str. Neff has been used to evaluate its capacity for lateral gene transfer (LGT) because it is considered one of the most important genomic evolution characteristics. Normally, phagocytic amoebae feed on microorganisms that share their same niche, including algae, bacteria, yeast, and viruses. When amoebas phagocytize, usually these organisms are degraded through phagolysosomes; however, there are instances of viruses and bacteria capable of surviving this process and achieving intracellular symbiosis with their host; these microorganisms are known as amoeba-resistant microorganisms (ARMs) [4]. In this section, we will first discuss ARMs of viral origin and then of bacterial origin.

From the phylogenomic analysis of this amoeba, interdomains have been predicted for LGT, finding 450 genes in the *Acanthamoeba* genome from this type of transfer, including genes originated from eukaryotic, bacterial, archaeal, and viral organisms. This demonstrates the extraordinary capacity of *Acanthamoeba* spp. to interact with diverse microorganisms while acting as a reservoir for a wide variety of species. Of the 450 genes mentioned earlier, 76 present homology with viral genes [14]. One of the predominant viruses among LGT of *Acanthamoeba* spp. are Megaviruses; Nucleocytoplasmic large DNA virus (NCLDV) being the most abundant virus found in metagenomic studies [52]. These have a genomic size range of hundreds of kilobases up to 2.5 Mb. Five phylogenetically related NCLDV clades, including giant viruses with a genome higher than 500 Kb, have been isolated in co-cultures with *Acanthamoeba* spp. [52]. However, after the discovery of Mimiviruses, another type of virus was identified: the virophages. These usually co-infect *Acanthamoeba* spp. cells, together with the Megaviruses, invading the transcription machinery used by the host-virus for its own subsistence, thus expanding the vision of viral variability in the environment [18, 54].

To analyze possible evolutionary changes of this FLA and to identify the possible functions caused by viral genetic material transfer, some phylogenomic approaches have been taken. Maumus and Blanc [52] managed to identify 267 LGTs that originated from giant viruses, 191 more genes than those initially reported by Clarke et al., [14], highlighting genes such as a major capsid protein, an ATPase, a viral-type RNA ligase, an endonuclease, a lipase type-3, an mRNA capping enzyme and a transposase [52]. The difference between the genes identified in both studies lies in the rapid advancement of sequencing technologies. The increasing number of genomes available in online databases allows for more complex analyses, expanding the knowledge on molecular biology and the genomic structure of these species. Interesting hypotheses have emerged from identifying viral origin genes found within the genome of *A. castellanii* str. Neff. First of all, in the case of genes that present transcriptional activity, it is suggested that they used to have a specific function in the virus and, subsequently, after integrating with the amoeba genome, acquired a new function inside the metabolism of this protist. These changes are probably related to the evolution of the *Acanthamoeba* genus. However, these genes without apparent transcriptional activity must have lost their function after experiencing mutations or deletions in coding regions as time went by, staying inside the genome as pseudogenes [52].

Another species used for the LGT analysis is *Acanthamoeba polyphaga* since virus lineages with an affinity towards infecting amoebae have been observed [12]. Such is the case of the Pandoraviruses and Pithovirus for *A. castellanii*, or the Faustovirus reported exclusively for *Vermamoeba vermiformis*. The Pandoraviruses are those that show major interactions with *A. polyphaga*; from the 366 genes of viral origin identified within its genome, approximately 32% correspond to homologs of these viruses. In a minor proportion, genes were found that originated from other viruses, such as Mimivirus (18%), *Mollivirus sibericum* (10%), Marseillevirus (7%), and *Pithovirus sibericum* (0.5%). Finally, through studies focused on comparative genomics done on *Acanthamoeba triangularis*, 99 ARM-origin genes were found, of which 35.4% showed homology with giant viruses. Similarly to *A. polyphaga*, most of these genes belong to Pandoravirus species (*P. quercus*, *P. inopinatum*, *P. macleodensis*, *P. neocaledonia*, and *P. salinus*). Additionally, homologs of *Medusavirus*, *Mimivirus*, *Tupanvirus*, *Catovirus*, *Marseillevirus*, *Pithovirus sibericum*, and *Mollivirus sibericum* were identified as part of the genomic structure of *A. triangularis* [32]. This extends considerably the repertoire of genes that *Acanthamoeba* species can integrate into their genome. For this reason, it begs the questioning about the relation between this genetic information transfer with evolution, of both the host amoeba and the viruses. The reason is that, despite all the advances in identification and classification of new viruses that metagenomics, comparative genomics, and pangenome analysis have brought us, our comprehension of the molecular biology, diversity, and evolution of these microorganisms is still scarce [25].

Another LGT mechanism of great interest to analyze in amoebas such as *Acanthamoeba* spp. is the one that occurs between ARMs of bacterial origin. Multiple genera have shown their survival inside the amoeba *in vitro.* Examples include bacteria such as *Mycobacterium tuberculosis*, *Chlamydia trachomatis*, *Rickettsia bellii*, *Burkholderia* spp*.*, *Coxiella burnetii*, *Escherichia coli*, *Francisella tularensis*, *Helicobacter pylori*, *Listeria monocytogenes*, *Porphyromonas gingivalis*, and *Vibrio cholerae* [4]. Previously, we mentioned 99 genes from ARMs described in the *A. triangularis* genome, 35.4% being of viral nature. The rest belong to ARMs of bacterial origin, which originated from LGT between the amoeba and intracellular bacteria. From this set of bacterial genes, 44 presented homology with *Chlamydia* spp., 16 with *Acinetobacter* spp. and *Pseudomonas* spp., and about two hypothetic proteins were related to *Legionella pneumophila*, all microorganisms known for their pathogenic capacity in humans [32]. It is possible that the great capacity of *Acanthamoeba* spp. to interact with all these kinds of bacteria resides in the presence of certain adhesion protein domains such as integrin and hemagglutinin of bacterial type described inside the genome of *A. castellanii* [14].

The genome of *A. castellanii* also codes for antiviral defense proteins, including the homologs of the main proteins from the NCLDV capsid and Dicer and Piwi homologs, related to the antiviral silencing mediated by RNA. Interestingly, this amoebae genome presents homologs of the interferon-γ-inducible lysosomal thiol reductase enzyme, a factor used by *L. monocytogenes* during macrophage infection and two GTPase homologs induced by interferon. These are known for their ability to activate cell-autonomous immunity, giving these cells the ability to eliminate infectious agents such as *Mycobacteria* and *Legionella* species. Additionally, a homolog of the natural resistance-associated macrophage protein described in the *D. discoideum* genome has been recognized. This protein is related to protection against *L. pneumophila* and *Mycobacterium avium* in macrophages [14]. The study described above is part of genomic analysis of great importance for the progress in understanding the behavior of *Acanthamoeba* spp. and its interactions with different kinds of microorganisms, as well as the molecular mechanisms needed for the description of its functional genomics.

Also, advanced genomic approaches have been used to propose a new detection system for *Acanthamoeba* spp. They employed PCR to obtain the pangenome, clustering orthologous genes belonging to the core genome, and used phylogenomic and phylogenetic analyzes derived from 14 representative genomes (BioProject PRJEB7687) and 33 isolates (environmental and clinical), respectively. The core genome was found to consist of 826 orthologs, of which only 15 showed significant divergences between the genomes. Finally, a gene encoding alanine-tRNA ligase was used to design a universal primer system for the *Acanthamoeba* genus, offering a more precise alternative identification system, unlike that used with 18S ribosomal sequences [11]. Another comparative genomics approach was performed between FLAs to identify the presence of peroxin orthologs, thus confirming the presence of peroxisomes in *A. castellanii*, *A. griffini*, *A. polyphaga*, *A. royreba*, and other amoebas such as *B. mandrillaris*, *N. fowleri*, and *N. lovaniensis*. This information that was very scarce in the field of research in opportunistic FLAs [28]. The importance of these organelles is related to their crucial role in various anabolic and catabolic pathways, changing their function depending on the organism that owns them [3].

Finally, Hasni et al. [32] identified virulence factors related to pathogenicity through a comparative genomics approach, comparing *Acanthamoeba* species such as *A. triangularis*, *A. castellanii* with other FLA (*N. fowleri*, *N. gruberi*, *N. lovaniensis*, *E. histolytica*, *D. discoideum*, and *W. magna*). This analysis reported 1004 orthologs within the *Acanthamoeba* genus. Of these, 48 were found to be related to *Acanthamoeba* keratitis (AK), mainly divided into two classes: factors that directly contribute to AK and factors that do so indirectly. Inside the group of direct factors, the most relevant are: a mannose-inducted protein that encodes for a protein necessary for the adhesion to the corneal surface; a total of 17 proteins related to cytoskeleton, including three acting-binding proteins; three phospholipases related to host cell lysis and membrane disruption; 11 peptidases that work to facilitate host invasion and a single glycosidase. During the analysis, it is noted that these virulence-related factors were not clustered in a common region within the genome of *A. triangularis*. These virulence factors are common ground with other pathogenic FLA, the mannose-binding protein used by the amoeba to mediate the adhesion with the host epithelial cells, proteases utilized for host evasion, and phospholipases for cell disruption and lysis as well as induction of inflammatory response. In the case of indirect virulence factors, the study mainly reported genes that code for heat shock proteins, vital for the survival of the pathogenic amoeba during infection [32].

Table 3 describes the general genome data between *Acanthamoeba* species corresponding to a BioProject (PRJEB7687) and those banked by the authors mentioned in this section. It is important to note that the BioProject data has been corrected in line with observations made by Fuerst [22], available on his website at Ohio State University https://u.osu.edu/acanthamoeba/genomes-of-acanthamoeba/. On this website, after an exhaustive analysis, he describes step by step the different motives behind why it is necessary to re-classify some genomes within the BioProject. Table 3 shows the difference of genome size between species, even within strains (example *A. castellanii* str. Neff and *A. castellanii* ATCC 50370). While it is well known that genome size diversity exists between all organisms, whether they come from evolution, gene transfer involving transposons, chromosomic mutations, or size change within repeated sequences [19], in the case of *Acanthamoeba* spp. data, it seems that in the majority, problems come from the need to improve the draft genomes. In the whole genome shotgun (WGS) Project of *A. castellanii* ATCC 50370, a total of 221,748 scaffolds were deposited, which serves as a sign of a highly fragmented genome. By contrast, the genome of *A. castellanii* str. Neff seems to possess a higher-quality assembly, or at least with lesser fragmentation (3192 scaffolds).

**Table 3:** Representative genomes of *Acanthamoeba* spp. available and collected from GenBank, and corrected through information provided by Fuerst (2020).

Organism	Strain	Sequencing technology	Genome size (Mb)	Assembly access number	Publication date	Reference
*A. astronyxis*	ATCC 30137	NA	83.4	GCA_000826245	January 2015	†
*A. castellanii*	ATCC 50370	NA	115	GCA_000826485	January 2015	†
*A. castellanii* str. Neff	ATCC 30010	Illumina GA IIx, 454 GS FLX Titanium and Sanger	42	GCA_000313135	January 2013	[14]
*A. culbertsoni*	ATCC 30171	NA	55.5	GCA_000826265	January 2015	†
*A. palestinensis*	ATCC 30870	NA	75.3	GCA_000826305	January 2015	†
*A. lenticulata*	ATCC 30841	NA	66	GCA_000826285	January 2015	†
*A. lugdunensis*	ATCC 50240	NA	99.4	GCA_000826425	January 2015	†
*A. mauritaniensis*	ATCC 50253	NA	106.8	GCA_000826465	January 2015	†
*A. triangularis*	ATCC 50254	NA	103.5	GCA_000826325	January 2015	†
*Acanthamoeba* sp*.*	ATCC 50496	NA	115.6	GCA_000826505	January 2015	†
*A. polyphaga*	ATCC 50372	NA	120.4	GCA_000826345	January 2015	†
*A. polyphaga*	Linc Ap-1	IonTorrent	49.35	GCA_001567625	February 2016	[43]
*A. quina*	ATCC 50241	NA	83.6	GCA_000826445	January 2015	†
*A. rhysodes*	ATCC 30973	N/A	75.8	GCA_000826385	January 2015	†
*A. royreba*	ATCC 30884	N/A	79.5	GCA_000826365	January 2015	†
*A. comandoni*	ATCC Pra 287	Ion Torrent PGM	86.2	GCA_002025285	March 2017	‡
*A. lenticulata*	ATCC 50704	Ion Torrent PGM	74.7	GCA_002105255	April 2017	‡
*A. triangularis*	ATCC 50254	Illumina MiSeq	66.4	*CACVKS010000000	March 2020	[32]
*A. pyriformis*	CR15	Illumina HiSeq 2500	683.1	**SRX2163158	January 2017	[72]

N/A = Information not available in BioProject placed in GenBank.

* No public data with this accession in GenBank.

** Transcriptome information only.

† Unpublished, BioProject created by University of Liverpool: https://www.ncbi.nlm.nih.gov/bioproject/PRJEB7687.

‡ Unpublished, created by AIT Austrian Institute of Technology: https://www.ncbi.nlm.nih.gov/assembly/GCA_002105255.1.

Additionally, this last genome includes useful data for users who wish to conduct comparative analyses, as it mentions the application of different sequencing technologies and a depth of coverage of 109.9X with Illumina reads. This parameter is considered good enough to establish an assembly with Illumina reads if a range of 60X–80X is met [19]. A brief explanation about the meaning of sequencing coverage could be resumed in “the number of times a genome has been sequenced” or “the total number of nucleotides within the reads is represented at least 60–80 times” [19, 38]. As we have mentioned before, the increase of genomic data requires the development of sophisticated bioinformatic resources. Sequencing coverage is important for the attainment of successful genomic assembly. However, there are other points to consider, such as the correct management of DNA, which must not contain any contaminant nor present degradation, and knowledge about the necessary algorithms to obtain an assembly [19, 26, 61], to name a few. Because of this, we consider it appropriate to suggest being more careful when analyzing genomes while aiming for comparative genomics, since a genome with a quality assembly is suitable for reliable gene annotation, and as a result, it allows the analyses to escalate in a specialized manner or to be bioinformatically advanced.

Although transcriptomics is not a highly developed field for these amoebae, there is a description of comparisons between the genetic expression by RNAseq of *A. castellanii* and *Entamoeba histolytica*, by analysis of orthological relationship and gene ontology, to understand their line of evolution and physiological restructuring. By grouping the orthologs between these two amoebas, a total of 1016 orthologs were observed, with 1357 homologous genes from *A. castellanii* and 1657 from *E. histolytica*. From this analysis, various groups of genes with the highest presence in *A. castellanii* were identified, among which the following stand out: proteins similar to the membrane resistance protein TolA, with 18 paralogs in *A. castellanii* and four in *E. histolytica*; highly expressed Rab 32 proteins, with eight derivatives in *A. castellanii* and three in *E. histolytica*; serine protease inhibiting proteins called Serpins, with six genes in *A. castellanii* and only one in *E. histolytica*. In contrast to *A. castellanii*, the groups with the highest presence in *E. histolytica* are transport proteins, beta-amylases, groups with the zinc-finger domain, maltose-acyltransferases, peroxiredoxins, endonucleases V, and phosphatases with 2C domain. Another finding worth mentioning is that *E. histolytica* appears to have more than twice the number of copies of genes coding for ribosomal proteins compared to *A. castellanii*, since *E. histolytica* has 213 copies, while *A. castellanii* only has 86 genes. Regarding genes related to pathogenesis, in axenic culture, *E. histolytica* maintained high expression of transcripts related to the formation of amoebapores A, B, and C. These genes are orthologs to saposins, lysosomal proteins that *A. castellanii* possesses, and are candidates for being the molecules responsible for cell lysis. In the same culture, *A. castellanii* only expressed one of the four saposins that it possesses. Also, concerning cell surface proteases, two were expressed by *E. histolytica* and only one by *A. castellanii*. Finally, the low expression of TMK96 kinase, a key element in the phagocytosis of the microorganism, of which no analog was found in *A. castellanii*, was identified [69].

After the gene annotation process of the *A. castellanii* genome, the presence of genes related to autophagy such as AcAtg8, an essential genetic activity for the amoeba’s survival during its cyst stage, was demonstrated. Given that the Atg8 proteins of *Saccharomyces cerevisiae* and *A. castellanii* share homologies, it was possible to identify the functionality of this protein in the recovery of autophagy from a strain of *S. cerevisiae* with its deficiency. This yielded a positive result in complementation experiments of the AcAtg8 sequence by plasmid pCM189 [65]. Also, a proteomic analysis involved in trophozoites has been carried out to identify surface proteins that are candidates for immunological detections with higher specificity. Through soluble protein fractions and others enriched with membrane proteins, their expression was obtained from LC-MS/MS spectrometry with peptides from SDS-PAGE. The presence of 503 proteins is reported; 308 proteins belonging to the soluble fraction, 119 proteins to the enriched one, and 79 were located in both fractions. Proteins from four categories of gene ontology (GO) were located; binding activity (GO: 0005488), catalytic activity (GO: 0003824), structural activity (GO: 0005198), and transport activity (GO: 0005215) [51]. Bouyer et al. [6] compared proteins expressed in trophozoite and cyst forms. In trophozoite form, the following were located: fructose-bisphosphate aldolase, transcriptionally controlled tumor protein, actophorin, and ribosomal protein S12, while in its encysted form, the enolase protein, HSP 70, and another involved in the gelation factor, which is responsible for maintaining the cellular form from its interaction with actin and serine protease of the subtilisin type.

Comparative proteomics was carried out to organize the outer membrane translocase complex (TOM) since it is recognized as a gateway for proteins to the mitochondria. It acts as the translocation manager of previously imported proteins, as well as the decoding of direction signals. Strains of *A. castellanii* and *D. discoideum* were used, managing to observe the level of organization of five subunits, TOM 22, TOM 20, TOM 7, TOM 70, and TOM 40, that show a similarity of their levels between species [73]. Also, the *A. polyphaga* proteome has been analyzed, concluding that when it is in the trophozoite stage, if it is kept in culture, it is slightly virulent; however, when passing the culture to a mammalian host, pathogenic traits are activated and tend to increase its virulence. It should be noted that proteins were found involved in survival mechanisms and protein renewal: HSP 90, HSP 20, HSP 70, HSP 91, and ubiquitin, among others [9].

### *Balamuthia mandrillaris*

Currently, there are only two drafts of the *B. mandrillaris* nuclear genome. The first 68 Mb genome was sequenced with PacBio technology from the strain V039 (GCA_001185145) and is described as a complex genome with hyperploidy [17]. The second genome, which was sequenced with Illumina from the strain 2046 (GCA_001262475), is 44.2 Mb in size. The alignment with BLASTn identified *A. castellanii* as the closest organism, with 4.3% identity. Prediction of ORFs has been carried out on this second genome, among which the following stand out: a truncated 5 Kb ORF with elements similar to a retrotransposon, the RNAse HI of the Ty3/Gypsy family, a reverse transcriptase, a chromodomain, and a retropepsin. Also, two 1.6 Kb ORFs were identified that were significantly aligned to an *E. coli* recombinase sequence. It was proven that this draft could be used as a reference to identify the presence of *B. mandrillaris* in a metagenomic analysis with next-generation sequencing (NGS). This is because in a study performed with the metagenome of a cerebrospinal fluid (CSF) sample taken from a patient with GAE, it was possible to detect nine specific readings for the amoeba [29]. Another case regarding the application of metagenomics was described by Kalyatanda et al., [42] in a patient with deteriorating health status and an undiagnosed condition. Metagenomics showed the presence of a “circulating microbial cell-free DNA” from *B. mandrillaris* in the patient’s plasma, allowing the physicians to initiate treatment with drugs recommended by the US Centers for Disease Control and Prevention (https://www.cdc.gov/parasites/balamuthia/treatment.html). This case suggests that this identification method is a fast and less invasive diagnostic tool in comparison to traditional techniques. Finally, the case of a pediatric patient diagnosed with tuberculosis and treated with empirical drugs was reported. As she did not improve with these treatments, a CSF sample was used to identify the pathogen using NGS, finding 165 reads corresponding to *B. mandrillaris.* These results were not consistent with the initial diagnosis using NGS, in which no DNA from bacteria, fungi, viruses, mycoplasma, or *M. tuberculosis* was observed. Unfortunately, both patients reported by Kalyatanda et al. [42] and the pediatric case mentioned above did not survive due to the late diagnosis of GAE, noting the need for the application of technology such as NGS for the identification of emergent pathogens such as *B. mandrillaris* and other FLAs specified in this review.

**Figure 2:**
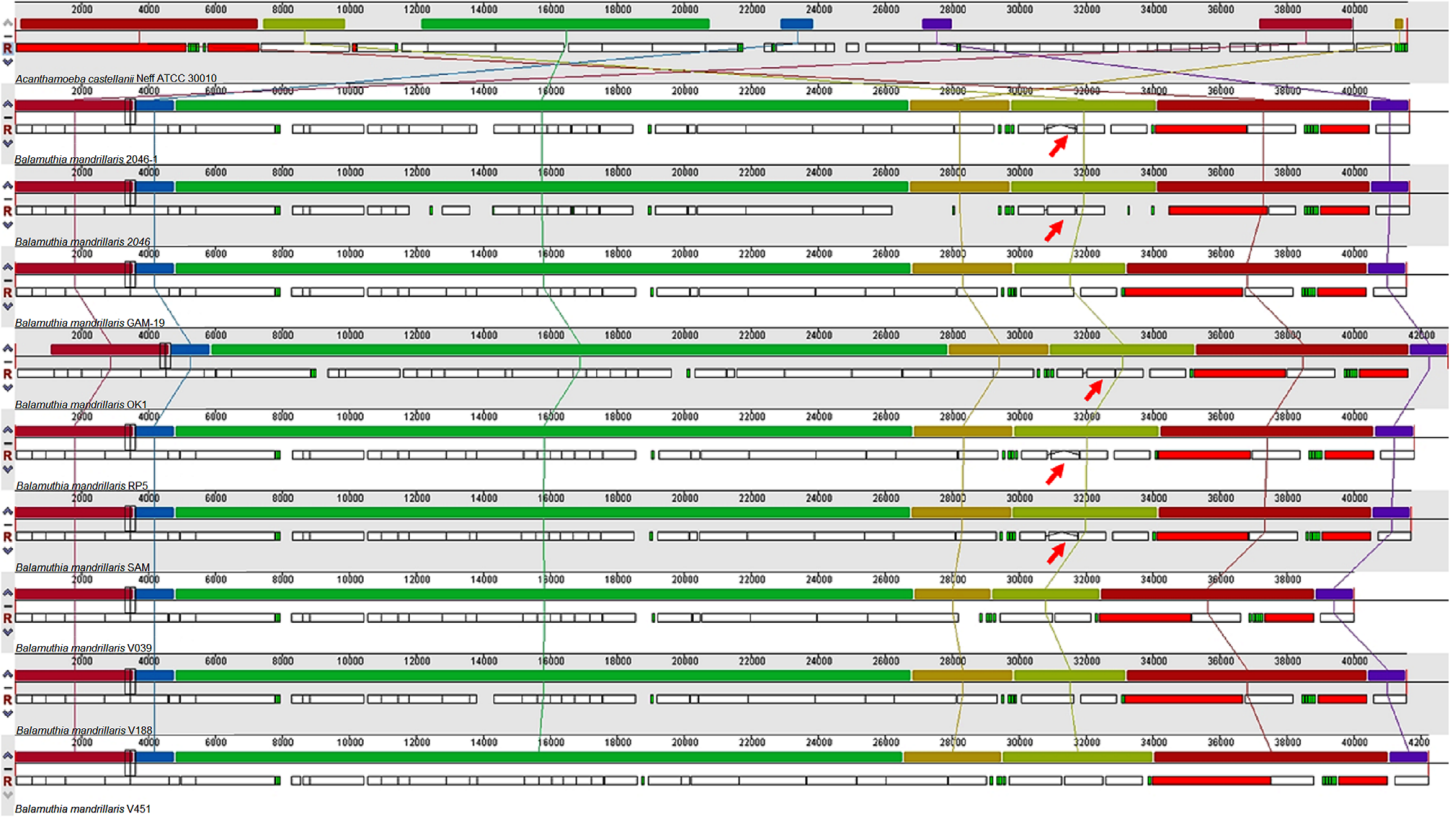
Alignment of *B. mandrillaris* mitochondrial genomes (mtDNA), using *A. castellanii* mtDNA as a reference. The lines show the synteny between the genomes. The upper rectangles show the locally collinear blocks and the lower boxes the annotated genes: red (rRNA), green (tRNA), and white CDS (coding sequence). Red arrows indicate the presence of the LAGLIDADG sequence disrupting the cox1 gene.

Although there is not much information on both the structural and functional annotation in the *B. mandrillaris* nuclear genome, the diversity of the amoeba mitochondrial genome has been sequenced and studied. This mitochondrial genome is around 41 Kb in size and has an adenine-thymine content of 64.8%. From the prediction of genes of this genome, two genes were obtained for rRNA, 18 for tRNA, and 38 coding sequences, with five sequences for hypothetical proteins. Furthermore, the *B. mandrillaris* mitochondrial genome had several syntenic blocks in common with *A. castellanii*. As a result, the analysis of synteny allows identification of the conservation of homologous genes and the order of genes between genomes of different species [49]. Several aspects are distinguished, such as the order of the rest of the coding blocks, the lack of a combined cox1/cox2 gene, and the absence of splicing of introns in the 23S ribosomal gene. In the case of the *B. mandrillaris* mitochondrial genome, the cox1 gene is interrupted due to an endonuclease ORF that contains the LAGLIDADG sequence, which also interrupts different genes in different strains (Fig. 2). Additionally, the putative gene for ribosomal protein s3 (rps3) is aligned with rps3 proteins from *E. coli* and *Thermus thermophilus* [29]. Regarding the similarity between different mitochondrial genomes, when comparing the genomes of seven different strains of *B. mandrillaris*, an average length of 41,526 bp and a nucleotide identity between 82.6 and 99.8% are distinguished. The most divergent strain was also found to be V451, due to an additional ORF of 1149 bp downstream of the cox1 gene. The second major cause of variation between genomes was putative introns because four of the seven sequences compared contain the ORF with the LAGLIDADG sequence mentioned above in the cox1 gene, while two of the three remaining mitochondrial sequences present this ORF in an insert of 790 bp in the 23S ribosomal gene. Similarly, a strain that is entirely devoid of this intron is known (strain V039). However, the ORF with the putative rps3 gene seems to vary in length among all the strains studied, and each one of the strains was found to have unique sequences for this gene. The possibility of using these disparities in the *B. mandrillaris* mitochondrial genome for genotyping in the future has been discussed. [29]. In Table 4, information on the mitochondrial genomes with the location of their variations is shown.

**Table 4:** *B. mandrillaris* mitochondrial genomes. Information from Greninger et al. [29], all the mtDNA was sequenced with the Illumina – MiSeq platform.

Strain	Location of LAGLIDADG	Genome size (bp)	GenBank accession numbers
2046	Cox1	41,656	KP888565
2046-1	Cox1	41,656	KT175740
V039	NA	39,996	KT175741
V451	23S	42,217	KT030670
OK1	Cox1	42,823	KT030671
RP-5	Cox1	41,784	KT030672
SAM	Cox1	41,707	KT030673
V188	23S	41,571	KT175738
GAM-19	23S	41,570	KT175739

A recent application of functional genomics performed by Phan et al. [63] obtained the first transcriptome of *B. mandrillaris* (strain V039) to reconstruct the proteome and identify the genes susceptible to 85 compounds with antimicrobial activity against this amoeba. From the *de novo* assembly, they found a higher percentage of similarity with *A. castellanii* str. Neff (45%) previously reported by Greninger et al. [29] when analyzing the mitochondrial genomes of *B. mandrillaris*. Additionally, it should be noted that with the transcriptome, supported by the draft genome of strain V039 deposited in GenBank, functional annotation of 80% of the proteins of this amoeba was done [63], finding 15 candidate proteins to be the target of drugs to fight infections like GAE. These proteins include mainly S-adenosyl-L-homocysteine-hydrolase, Histone deacetylase 1, HSP90-alpha, Exportin-1, and some copies of DNA topoisomerase II.

## The omics era and the future of pathogen characterization

Almost 20 years have passed since the introduction of the first commercially available NGS platform. Since then, the genomic and post-genomic era has significantly expanded our understanding of structural and functional genomics as well as the genetics of many diseases. Subsequently, the creation of the concepts of “omics” (transcriptomics, genomics, proteomics, metabolomics, etc.) further expanded new knowledge about various organisms and how they relate to their environment and the response to variable conditions. The baseline for research built on multiple omic approaches is the assembly of a high-quality genome, since functional genomics works as a baseline for the others. The main objective of most research papers related to pathogens is to identify either their pathogenic mechanisms or their metabolic processes to develop a treatment able to inhibit their ability to cause disease. A complete, annotated genome allows the prediction of factors, such as proteins or regulator RNAs, and identification of patterns in full scale. In contrast to traditional molecular biology that must work on smaller sets of genes to correlate, actual genomics allows for the correlation of the global picture. Integration of genomics with other approaches, such as transcriptomics or metabolomics, broadens the spectrum. This makes it possible to study the relation of the whole genetic picture with either the specific transcripts a pathogen produces in a set timeframe under certain conditions in the case of transcriptomics, or with metabolomics it is possible to relate the full genetic set from the genome with itself to discern or understand metabolic routes and their possible related metabolites. When adding proteomics to the mix, this integration of multi-omic tools is known as proteogenomics. This approach is able to identify both novel and estimated protein sequences involved in an organism’s life cycle at full scale, tracking any potential factor from its genetic source to its related transcript and ultimately its final form, protein or not, as well as their development and relation to any metabolic route.

The number of infections reported by free-living amoebae has increased in recent years. Knowing about the pathogenic mechanisms of *N. fowleri*, *B. mandrillaris*, and *Acanthamoeba* spp. is essential since most cases are identified *post-mortem* due to the lack of timely and effective treatments for infections (PAM, GAE, and AK). Fortunately, the FLA research area is developing faster thanks to the applications of omic technologies, which, integrated with bioinformatic strategies and specialized software development, leads to a better understanding of pathogenesis, pathogenomics, and virulence factors in the process of improving identification systems, designing targeted treatments and, consequently, reducing the high percentage of mortalities presented by these pathogens.

In the future, full-scale projects involving proteogenomics will most certainly be able to identify specific virulence and metabolic factors and the full mechanisms where they interact, to develop and study more effective and precise medication and treatment options. Even then, this does not mean the end of other techniques used today. As presented before, cultivation techniques such as the passage of *N. fowleri* trophozoites through mouse specimens have proven to develop highly virulent amoebae, optimal for the identification of virulence factors for both traditional molecular techniques and full-on transcriptomics. Furthermore, the application of multiple omic techniques is still very limited due to certain factors. One is the delicate nature of the sampling process and full genetic material extraction. Others include the practically mandatory requirement of knowledge in bioinformatics basics and strategies to process and analyze data, and the high costs of both the instruments used to obtain the sequences and the machines to process them. And even with the application of these highly demanding techniques, the possible results obtained by their use will still need to be confirmed via standard molecular techniques for them to be plausible. As a result, both molecular and cultivation techniques used today will remain relevant in the near future when more complete omic approaches are used in FLA research. Unquestionably, the application of omic sciences to FLA research is a field in the exploration process, with great potential to elucidate genes and their molecular mechanisms, which will take more ground in future research. However, it is important to improve the already sequenced genomes in order to make quality gene annotations and support them with transcriptomic data. The aim is to obtain more precise genetic information, as well as to adapt current cultivation and molecular techniques used in the study of certain FLA to the rest of them to acquire all the benefits of ideally conditioned cells for the application of omic approaches.
